# Integrating genomic information with protein sequence and 3D atomic level structure at the RCSB protein data bank

**DOI:** 10.1093/bioinformatics/btw547

**Published:** 2016-08-22

**Authors:** Andreas Prlić, Tara Kalro, Roshni Bhattacharya, Cole Christie, Stephen K. Burley, Peter W. Rose

**Affiliations:** 1 RCSB Protein Data Bank, University of California San Diego, San Diego Supercomputer Center, La Jolla, CA 92093, USA; 2 RCSB Protein Data Bank, Department of Chemistry and Chemical Biology, Center for Integrative Proteomics Research, Institute for Quantitative Biomedicine, and Rutgers Cancer Institute of New Jersey, Rutgers, The State University of New Jersey, Piscataway, NJ 08854, USA; 3 Bioinformatics and Medical Informatics, San Diego State University, San Diego, CA 92182, USA

## Abstract

**Summary:** The Protein Data Bank (PDB) now contains more than 120,000 three-dimensional (3D) structures of biological macromolecules. To allow an interpretation of how PDB data relates to other publicly available annotations, we developed a novel data integration platform that maps 3D structural information across various datasets. This integration bridges from the human genome across protein sequence to 3D structure space. We developed novel software solutions for data management and visualization, while incorporating new libraries for web-based visualization using SVG graphics.

**Availability and Implementation:** The new views are available from http://www.rcsb.org and software is available from https://github.com/rcsb/.

**Contact:** andreas.prlic@rcsb.org

**Supplementary information:** Supplementary data are available at *Bioinformatics* online.

## 1 Introduction

The Protein Data Bank (PDB; http://wwpdb.org) was established in 1971 as the first open access digital resource in biology with just seven X-ray crystal structures. The global PDB archive now contains more than 120,000 experimentally determined three-dimensional (3D) atomic level structures of biological macromolecules, all of which are freely accessible without limitations on usage *via* the Internet. The Research Collaboratory for Structural Bioinformatics Protein Data Bank (RCSB PDB) is the US regional data center for the Worldwide PDB (wwPDB; wwpdb.org), which manages the global PDB archive. The RCSB PDB website (rcsb.org) is built atop a data warehouse that integrates ∼60 external resources (Rose *et al.*, 2014).

It is not unusual for PDB entries to provide atomic level structural data that covers only part of the full-length protein polypeptide chain. For technical reasons, it is not always possible to determine 3D structures for every polypeptide chain segment in a protein of interest. In order to provide a more insightful representation of how a PDB entry relates to its full-length UniProtKB protein sequence (The UniProt Consortium, 2014), and, going further, how a PDB entry relates to the gene encoding for that protein, we developed the *RCSB Protein Feature View* and *Gene View*.

Integration of underlying data is an important consideration when developing rich views of biological data. To enable these new views of PDB archive data, we developed novel software components for mapping genomic data to protein sequence isoforms and 3D protein structures, and for managing local installations of UniProtKB. Herein, we are making these various software components available as re-usable open-source libraries, reflecting the enduring commitment of the RCSB PDB to best practices in open-source scientific software development (Prlić and Procter, 2012). For a comparison with existing approaches, please see Supplementary Table 1.

## 2 RCSB PDB protein feature view

**Fig. 1.**
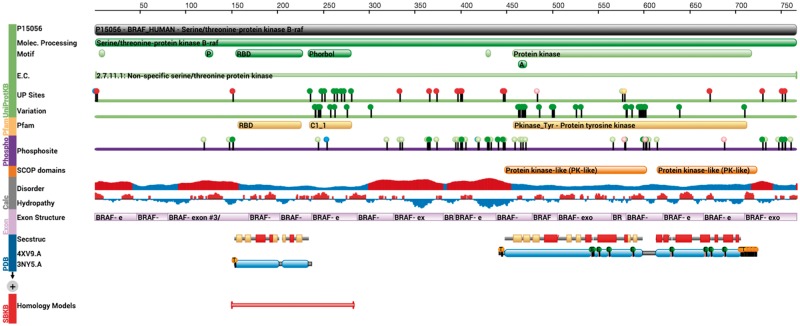
RCSB PDB Protein Feature View. This view provides a graphical summary how PDB data and external annotations are related to UniProtKB sequences. Here, the sequence of BRAF is shown. For a description of the data-tracks, see main text

The *RCSB Protein Feature View* combines annotations from various data sources in the context of a reference UniProtKB sequence, providing comparisons of structures represented in PDB archive entries (Fig. 1). A color-coded, graphical representation of these data indicates the provenance of a data ‘track’, e.g. green bar indicates all information coming from UniProt, while PDB derived data is denoted in blue.

*Protein Feature View* comes in two flavors; a *simplified version* that shows how a single PDB entry relates to a UniProtKB sequence is available on each RCSB PDB Structure Summary page. A *detailed version* shows how all PDB entries for a single UniProt sequence relate to each other and the reference sequence. *Protein Feature View* includes information from the wwPDB Validation Reports (*simplified version* only) (Read *et al.*, 2011), together with manual annotations provided by wwPDB Biocurators, identifying any sequence mismatches detected when comparing the protein sequences of PDB entries and UniProt reference sequences.

Some such sequence mismatches arise because of the presence of expression tags, engineered mutations, chromophores, phosphorylation and other types of sequence modifications, all which are depicted using custom icons.

A new 3D visualization is available that supports mapping of any protein sequence annotation from the *Protein Feature View* onto protein structures using the NGL viewer (Rose and Hildebrand, 2015). The data integration enabled by the tools described below is used to provide several novel tracks: The *Exon Structure* track shows a mapping of the exon/intron boundaries onto the first (canonical) isoform sequence, as provided by UniProt. The *Variation* track indicates the position of genetic variations, as annotated by UniProt. Protein phosphorylation annotations are loaded from the PhosphoSitePlus database (Hornbeck *et al.*, 2015). *Protein Feature View* also shows genetic variant annotations, as derived from UniProtKB. Pfam (Finn *et al.*, 2015) annotations are re-computed weekly using an automated pipeline connected to the HMMER 3 web-service. Parsers for all of the data described above have been made available as part of BioJava, a widely used open-source library in the Java programming language (Prlić *et al.*, 2012).

For the user-frontend, the *Protein Feature View* JavaScript library employs a simple RESTful server-client communication protocol. The RCSB PDB web site provides the data ready for visualization as JSON data objects (created using BioJava). The JavaScript layer then visualizes the JSON data using SVG graphics. JavaScript library source code is publicly available from https://github.com/rcsb/proteinfeatureview.

## 3 UniProt object-relational mapping

*Protein Feature View* displays annotations derived from PDB together with annotations provided by UniProt. Comparison of complementary data provided by these two primary databases enables novel insights into publicly available scientific information.

UniProt provides functional annotations, including domain organization, oligomeric structure and catalytic activity (if available) of proteins. The header section of the *Protein Feature View* provides a textual description of these annotations from UniProt. If references to other UniProt entries are identified within the UniProt textual descriptions, we provide links for easy navigation between and among entries.

Operation of *Protein Feature View* by the RCSB PDB, requires that we host a complete in-house copy of the SwissProt subset of UniProt (the manually annotated, high quality subset of UniProt), plus parts of TREMBL that map to PDB entries. We developed a new tool that allows easy autogeneration of a local copy of UniProt within a relational database. This approach uses the UniProt XML schema to automatically generate a database mapping with object-relational Java classes. Our tool can be used to load any UniProt XML file into a relational database, where it becomes possible to perform queries across all of UniProt. For the RCSB PDB *Protein Feature View*, we are currently maintaining a regularly updated, local installation of ∼550k SwissProt and TREMBL entries. Source code is for overnight data loading is available from https://github.com/rcsb/uniprot-or-mapping.

## 4 Mapping genomic data to 3D protein structure

Human proteins in the PDB are of special interest to the biomedical research community. We have, therefore, built a data display system that connects human genes in chromosomes to 3D structures of human proteins in the PDB archive (http://www.rcsb.org/pdb/chromosome.do). Again, built atop BioJava, we developed a pipeline that maps human genes, as available from the HGNC (Gray *et al.*, 2015) (www.genenames.org) and the UCSC gene browser (Rosenbloom *et al.*, 2015) to UniProt and to the PDB, using the new UniProt representation described in the previous section.

When comparing alternative transcripts for genes with UniProt, we find that the canonical isoform, typically represented as the default in UniProt, does not always correspond to the genomic annotation. Our mapping pipeline identifies the UniProt isoform corresponding to the each of the human protein structures found in the PDB. This mapping can also be done in the reverse direction, allowing any amino acid to be mapped to its corresponding codon.

Once the correct mapping to UniProt has been established, we then project from UniProt to PDB using the SIFTs mapping framework, which provides a residue level mapping between protein sequence and 3D structure (Velankar *et al.*, 2013). SIFTs mappings are currently computed relative to the canonical (usually the longest) UniProt isoform. An additional alignment step allows us to map the genomic coordinate *via* the correct splice isoform, to the canonical isoform used by SIFTs, and finally to the 3D protein structure.

Our newly developed database contains annotations for ∼19,000 human genes. With RCSB PDB analysis tools, any genomic location can be mapped to UniProt and the PDB archive of 3D structures (whenever a mapping is possible). At present, ∼5100 distinct human genes can be mapped onto ∼7900 PDB entries. Going forward, this data mapping strategy will be extended to include calculated homology models of human protein structures, which will significantly increase the number of human genes that can be linked to 3D atomic level structural information.

Mapping from genome to protein sequence to 3D protein structure is not trivial. We have, therefore, both made the source code for our system available (https://github.com/rcsb/uniprot-or-mapping) and provided a web-form, wherein genomic coordinates of the human genome can be mapped to protein sequences and 3D structures (whenever possible). The web-form can be used to confirm intron/exon boundaries in proteins, and map single-nucleotide variations (SNVs) or any other positional data onto protein annotations in 3D. Our mapping tools integrate the RCSB PDB Gene View (see below) with the *Protein Feature View*, allowing us to combine user-provided human genome coordinates with available protein functional annotations and 3D atomic level protein structure using the *PV* protein viewer https://github.com/biasmv/pv (doi: 10.5281/zenodo.20980), an alternative to the popular 3D viewer JSmol (Hanson, 2010).

Mapping of PDB entries to the human genome is available from http://www.rcsb.org/pdb/browse/homo_sapiens.do .

## 5 RCSB PDB gene view

To visualize the relationship of genomic and 3D structural data, we created a *Gene View.* This tool allows browsing of the human genome with PDB data highlighted onto corresponding genomic ranges. Similar to the *Protein Feature View*, these data can be correlated with other genomic and protein functional annotations, such as gene structure annotations, DNA repeats, or sequence conservation, across ∼50 vertebrate genomes, which are shown using the same ‘data-tracks’ approach, that is also used by the *Protein Feature View*. The *Gene View* interoperates with other genome databases, such as Ensembl (Yates *et al.*, 2016). The graphical view is built atop the BioDalliance genome browser (Down *et al.*, 2011). Users can scroll and zoom around any human gene. Dragging title boxes will reorder data tracks. To represent the region on a chromosome where a certain gene is located, we create a new ideogram representing the chromosomal karyotype (https://github.com/rcsb/karyotypeSVG).

By searching for human gene names in the top-bar search of the RCSB PDB, it is possible to access *Gene View*. In addition, as with the *Protein Feature View*, it is also available from the ‘Macromolecules’ section of the Structure Summary page for human proteins.

## Supplementary Material

Supplementary Data
