# Supplementary material for: Integrating genomic information with protein sequence and 3D atomic level structure at the RCSB protein data bank
Source: Bioinformatics. 2016 Aug 22;32(24):3833–5. doi: 10.1093/bioinformatics/btw547 (PMC5167066; doi:10.1093/bioinformatics/btw547)
Supplement: Supplementary Data [file supp_32_24_3833__index.html]

Integrating genomic information with protein sequence and 3D atomic level structure at the RCSB protein data bank — Integrating genomic information with protein sequence and 3D atomic level structure at the RCSB protein data bank — Supplementary Data 

# Integrating genomic information with protein sequence and 3D atomic level structure at the RCSB protein data bank

## Supplementary Data

files

- Supplementary Data - zip file
